# Detection of large areas of thin-cap fibroatheroma in a recurrent STEMI patient using a novel artificial intelligence algorithm: moving from 2D to 3D

**DOI:** 10.1093/eurheartj/ehaf189

**Published:** 2025-04-08

**Authors:** Thijs J Luttikholt, Jos Thannhauser, Niels van Royen

**Affiliations:** Department of Cardiology, Radboud University Medical Center, PO Box 9101, 6500 HB Nijmegen, The Netherlands; Department of Cardiology, Radboud University Medical Center, PO Box 9101, 6500 HB Nijmegen, The Netherlands; Department of Cardiology, Radboud University Medical Center, PO Box 9101, 6500 HB Nijmegen, The Netherlands

Optical coherence tomography (OCT) is a valuable imaging tool in percutaneous coronary intervention (PCI), recommended for stent guidance and evaluation.^1^ Moreover, OCT-imaging can visualize high-risk plaques, such as thin-cap fibroatheroma (TCFA), which have prognostic value.^2,3^ However, manual OCT-interpretation is time-consuming, subject to interobserver variability^4^ and, most importantly, assesses TCFA in a two-dimensional, single-frame manner. It is likely that prognosis depends on TCFA-extent rather than presence alone, similar to lipid burden in near-infrared spectroscopy.

Our group developed OCT-AID, an artificial intelligence (AI)-algorithm for OCT-segmentation (Supplementary data online, *Video S1*) and plaque characterization.^5^ OCT-AID enables automated quantification of TCFA-area, as demonstrated in the present case.

A 75-year-old woman presented with ST-elevation myocardial infarction (STEMI) due to an occluded LCx, which was successfully treated with PCI. A non-culprit lesion (fractional flow reserve = 0.84) in the LAD was left untreated (*Panel A*). Three years later, she was readmitted with an anterior STEMI and LAD-occlusion at the site of the previously interrogated lesion (*Panel B*).

During the first event, OCT was performed of the LAD as part of the PECTUS-obs study.^2^ Conventional OCT-imaging showed a TCFA-positive plaque (*Panels C* and *D*). AI-driven segmentation (OCT-AID) identified extensive thin-cap regions with 170 frames (31%) containing TCFA. The AI-generated lipid plot (*Panel E*) visualizes lipid distribution and fibrous cap thickness (FCT) across 360 single-degree angles per OCT-frame. Total thin-cap area (FCT < 100 μm) was 0.11 mm^2^, while TCFA-area (FCT < 65 μm) measured 0.022 mm^2^, representing 8.9% and 1.8% of the total lipid area, respectively.

AI-tools now enable real-time quantification of TCFA-area. The present case suggests that TCFA-area, rather than single-frame TCFA-presence, is a potential prognostic marker for recurrent events.

**Figure:**
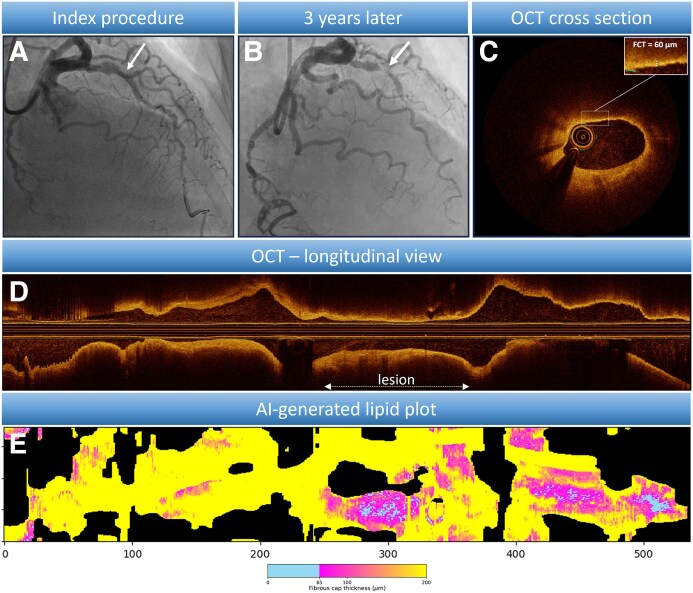


Supplementary data are available at *European Heart Journal* online.

N.v.R. declares grants from Koninklijke Philips NV, Biotronik, Medtronic Inc., and Abbott; speaker fee from MicroPort, Bayer AG, and RainMed Medical.

The data underlying this article are available from the corresponding author upon reasonable request. The code and annotated data underlying this manuscript cannot be made publicly available due to funding source restrictions.

This publication is part of the project ROBUST: Trustworthy AI-based Systems for Sustainable Growth with project number KICH3.LTP.20.006, which is (partly) financed by the Dutch Research Council (NWO), Abbott, and the Dutch Ministry of Economic Affairs and Climate Policy (EZK) under the programme LTP KIC 2020-2023. Funding partners were not involved in the collection, analysis, and interpretation of the data, and in the preparation, review, or approval of the manuscript. All content represents the opinion of the authors, which is not necessarily shared or endorsed by their respective employers and/or sponsors.

## Supplementary Material

ehaf189_Supplementary_Data
